# Combining Stress Speckle Tracking with High-Sensitivity C-Reactive Protein in Diagnosis of Coronary Artery Disease

**DOI:** 10.3390/jcdd9050123

**Published:** 2022-04-20

**Authors:** Ahmed M. Saleh, Konstantin Zintl, Johannes Brachmann

**Affiliations:** 1 Cardiology Department, Klinikum Westmuensterland, St. Agnes Hospital, 46397 Bocholt, Germany; 2 Cardiology Department, Klinikum Coburg, 96450 Coburg, Germany; ziko108@web.de; 3 Regiomed Kliniken, University of Split School of Medicine, 96450 Coburg, Germany; johannes.brachmann@regiomed-kliniken.de

**Keywords:** atherosclerosis, cardiovascular diseases, inflammation, cardiac imaging, speckle tracking

## Abstract

Introduction: CAD (coronary artery disease) is a leading cause of death and disability in developed nations. Exercise testing is recommended as a first-line diagnostic test for patients with stable angina pectoris. In addition to myocardial strain, high-sensitivity CRP (hs-CRP) can predict the presence of significant coronary artery disease. Aim of work: The purpose of this study was to demonstrate the utility of 2D-speckle tracking at rest and under stress along with hs-CRP for detection of CAD in patients who were referred to the chest pain unit with stable or low risk unstable angina pectoris. Methods: A total of 108 individuals met the inclusion criteria and gave their written consent to participate in this study. Coronary angiography was performed within 48 h after admission to the chest pain unit. Myocardial strain was recorded at rest and during dobutamine administration. Results: Global longitudinal strain at stress appeared to be moderately correlated with the presence of significant coronary artery disease (CAD); r = 0.41, *p* < 0.0001. A moderate correlation was also found between global longitudinal strain at stress and the severity of coronary occlusion; r = 0.62, *p* < 0.0001. With a cut-off value of −19.1, global longitudinal strain under stress had a sensitivity of 74.1% and a specificity of 76.7% for detecting significant CAD. Hs-CRP was significantly higher in patients with manifested CAD. Conclusion: Evaluation of longitudinal strain parameters at rest and under stress may predict coronary artery disease in patients with stable angina pectoris. A measurable Hs-CRP is a potential marker of coronary stenosis. Strain data could assist in diagnosing CAD severity.

## 1. Introduction

Atherosclerosis of the epicardial coronary arteries results in coronary artery disease. This reduction in coronary artery flow may occur asymptomatically or symptomatically, may be associated with exercise or rest, or may cause myocardial infarction or angina, depending on the severity of involved coronary obstruction and the rapidity of development [1].

One of the established tools for diagnosing coronary artery disease (CAD) is dobutamine stress echocardiography (DSE). Due to relatively low interobserver agreement and the qualitative nature of the diagnosis, it is difficult to achieve high accuracy of visual diagnosis of wall motion abnormalities during DSE [2,3]. Though DSE is being standardized with wall motion scores and enhanced myocardial border detection with transpulmonary contrast, the learning curve is steep and variable reproducibility between observers and institutions still persists [4,5].

These limitations can be overcome by measuring the global longitudinal strain (GLS) and strain rate of the myocardium. Echocardiographic longitudinal strain reliably indicates regional myocardial deformation and deformation rate (strain rate, SR). During acute and chronic ischemia, as well as stress-induced ischemia, the motion of the left ventricular wall is accurately depicted. The use of myocardial strain during DSE to assess viability and ischemia has recently been reported [6,7,8,9].

As atherosclerosis progresses, inflammation plays a vital role in plaque stability or rupture. Studies have shown a correlation between elevated high-sensitivity C-reactive protein and coronary stenosis and severity of stenosis [10,11].

In this study, we examined the utility of myocardial strain derived from dobutamine echocardiography under rest and stress in relation to invasive coronary angiography results in patients who presented with acute chest pain.

## 2. Methods

**Figure 1:**
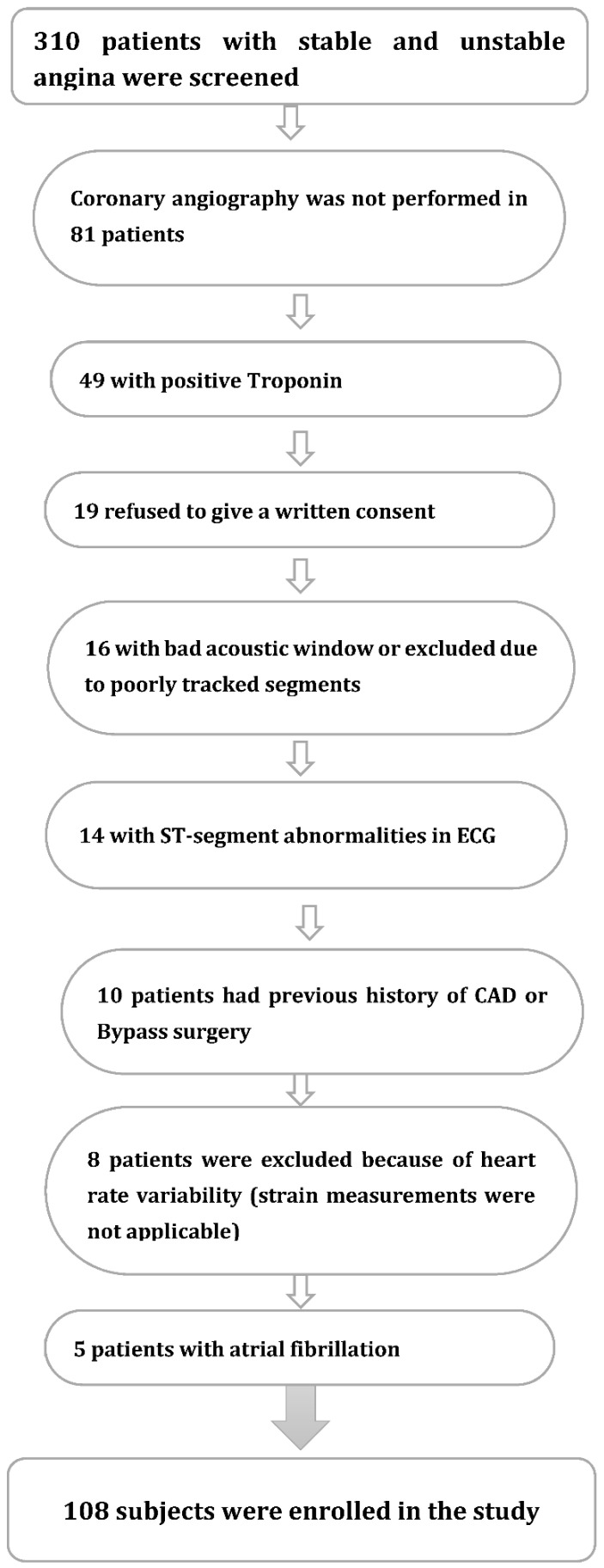
Flow chart of patients enrolled in the study and reasons for exclusion.

Patients with typical chest pain were admitted to the chest pain unit (CPU) at Coburg Hospital, Germany. Three hundred and ten patients with stable and unstable angina (TIMI risk score 0–1) were screened. Among them, 108 individuals matched the inclusion criteria and gave their written consent to be enrolled in this study (Figure 1). All included subjects were older than 18 years and suited for stress testing. Coronary angiography was performed within 48 h of admission to the chest pain unit. For the purposes of this analysis, we characterized significant coronary stenosis as ≥70% luminal obstruction. We opted to use a widely accepted standard for evaluating angiographic significance, even if less severe stenosis might be associated with risk for cardiovascular events. Venous blood samples were collected from all patients. As part of our laboratory’s examination, the hs-CRP was measured by enzyme-linked immunosorbent assay.

An elevated troponin level, ST-segment elevation, or depression during the admission process at CPU, a history of coronary artery disease or acute myocardial infarction, coronary artery bypass grafting, chronic total coronary occlusion, significant valvular heart disease, end stage renal failure, or refusal to give the written consent were considered exclusion criteria.

Prior to invasive assessment with coronary angiography, a dobutamine stress echocardiogram was performed.

### 2.1. Echocardiography

Examinations were performed with a digital ultrasonic device system (Vivid 9, GE Vingmed Ultrasound, Horten, Norway) in harmonic mode 2.0/4.3 MHz with maximal frame per second (FPS) count available at the necessary sector width. The range of FPS was from 64 to 112 with a mean value of 83.

Nevertheless, conventional echocardiography measurements were performed, including 2D measurements of the cardiac chambers and the Ejection Fraction (EF%), continuous wave and pulsed wave Doppler studies, color Doppler studies, Simpson’s method for calculating the Ejection Fraction, and analysis of wall motion abnormalities.

### 2.2. Dobutamine Echocardiography

During DSE, dobutamine was infused directly into the bloodstream at a dose of 10 mg/kg/min via a peripheral infusion line. The dose was increased at 3-min intervals to 20, 30, and 40 mg/kg/min with intravenous atropine up to 2 mg given, if necessary, to augment the heart rate response. Blood pressure and electrocardiogram were monitored continuously.

The following criteria were considered for termination of the test: 85% of the age-predicted maximum heart rate response, development of wall motion abnormality, severe electrocardiographic changes indicative of angina, systolic blood pressure greater than 240 mm Hg, abnormal blood pressure reaction during stress, or significant arrhythmia.

Two experienced echocardiographers recorded images in the lateral decubitus position. Standard 2D grayscale images of three standard apical views (four-chamber, two-chamber, and apical long-axis) and parasternal long-axis and parasternal short-axis views at the level of mitral valve, papillary muscles, and apex were acquired at rest, at a dobutamine dose of 20 mg/kg/min, at peak stress, and at recovery 1 min after stress. As per protocol, a cine image of one representative cardiac cycle per stage and view was digitally stored for later offline analysis.

In order to achieve optimal speckle-tracking at high heart rates, each image was optimized for left ventricular analysis, and the picture frame rate was increased to reach a target of 90 frames/s without compromising endocardial border detection. Two experienced echocardiographers, blinded to other results, examined wall motion visually. At the end of enrollment, the operators evaluated 20 random studies again to assess intra- and interobserver variation.

### 2.3. Quantification of Strain Measurements 

Echocardiographic images were obtained prior to coronary angiography. Three uninterrupted cardiac cycles were applied for each of three standard apical (two-, three-, and four-chamber) views and were kept for offline longitudinal strain analysis, using EchoPAC software (GE Ultrasound, v10.8.1). For assessment of longitudinal strain, we recorded standard 2D ultrasound images with a frame rate between 60 and 90 frames per second (fps) from the standard views. The endocardium was manually marked out from selected cineloops of apical view images. A further manual adjustment of the region of interest was applied after visual evaluation. The full image was excluded if >2 segments were poorly tracked. Speckle tracking was carried out on all three apical views of rest images and negative global systolic longitudinal strain was estimated. The average of GLS in apical four-, three-, and two-chamber images was used for our analysis. 

### 2.4. Statistical Analysis 

Our statistical analyses were conducted using SPSS 21.0 (SPSS Inc., Chicago, IL, USA). Continuous variables are expressed as mean ±standard deviation and categorical variables are expressed as counts and %. We calculated diagnostic measures including sensitivity, specificity, positive predictive value (PPV), and negative predictive value (NPV). In order to compare the diagnostic performance of myocardial strain between rest and stress conditions, receiver operating characteristic curves were analyzed. The correlation between variables was assessed using Pearson’s correlation coefficient. In this study, values of intra- and inter-observer reproducibility were evaluated by intra-class correlation coefficients (ICC). In all analyses, *p* < 0.05 was considered statistically significant. 

## 3. Results

**Figure 2:**
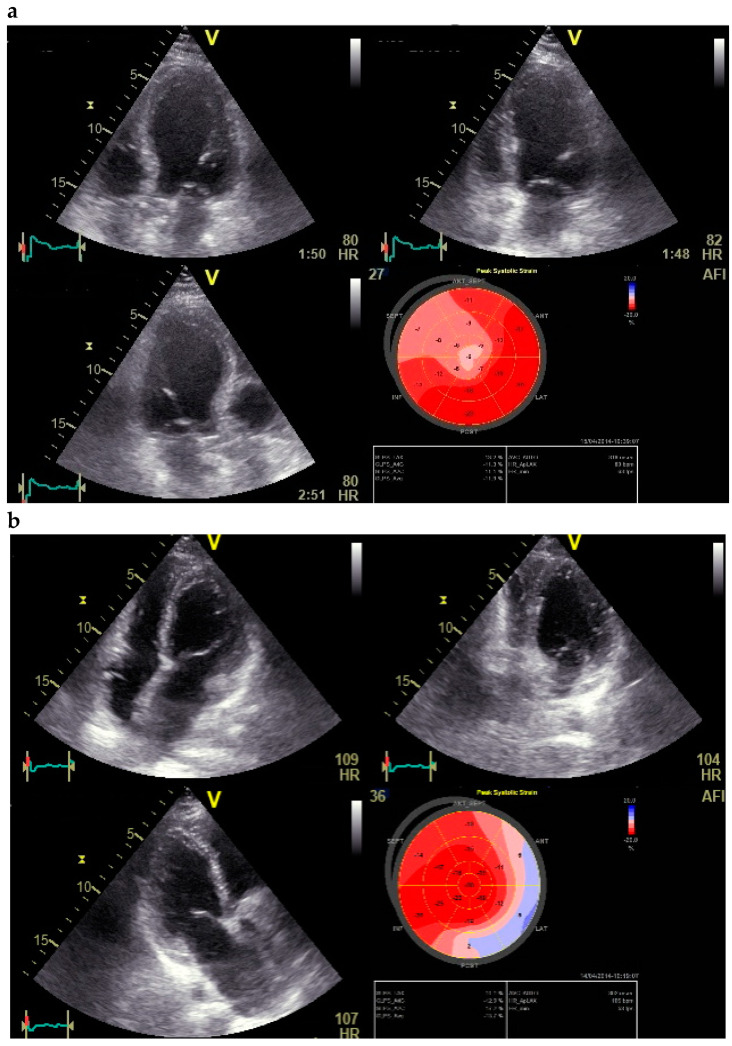
(**a**) Global longitudinal strain (GLS) by speckle tracking at rest showing decreased strain in septal region. (**b**) GLS by speckle tracking under stress (first phase stress at 20 mg/kg/min intravenous infusion of dobutamine) of the same patient showing decreased strain distributed over anterior and anterolateral region (significant left circumflex stenosis was revealed in coronary angiography).

A total of 108 patients who presented to our chest pain unit with stable and unstable angina pectoris for coronary angiography were examined. Subjects excluded from the study are identified in Figure 1. Diagnoses were based on clinical presentation, ECG results, and laboratory findings. In order to determine the global longitudinal strain values, dobutamine stress echocardiography was performed prior to invasive evaluation (Figure 2). 

### 3.1. Demographic Features

Women accounted for 50% of our study cohort (54). A total of 87 of the patients were hypertensive while 52 had already been diagnosed with diabetes. A total of 41 patients of the studied group were tobacco smokers (38%) and 40 patients showed either elevated lipid profile or were taking lipid-lowering drugs.

We found that the average age of our cohort was 64 years old ± 10 and that the mean body mass index was 25.4 kg/m^2^ ± 3.8. Troponin levels at admission were 0.081 ng/mL ± 0.03. Of our subjects, 35 had coronary artery lesions > 70% on coronary angiography (Table 1).

A significant increase in hs-CRP was observed among patients with significant CAD (3.2 mg/L + 1.8) as compared to those without CAD (1.9 mg/L + 1.5). The -vessel CAD patients showed a mean hs-CRP value of 4.9 + 1.1 mg/L. To detect significant coronary stenosis, a cut-off value of 2.8 mg/L had 85.7% specificity (95% CI: 75.9–92.6) and 67.7% sensitivity (95% CI: 48.6–83.3).

A total of 31 patients showed segmental wall motion anomalies in response to dobutamine stress (at least in one segment). Patients with CAD had significantly lower global longitudinal strain (GLS) at rest than those without (−16.9% + 2.8 vs. −18.4% + 3.1; *p* = 0.001). Additionally, GLS under stress was lower in people with significant CAD (−21.9% + 3.7 vs. −20.8% + 3.3).

We found a weak correlation between WMA under stress and CAD, r = 0.26, *p* < 0.05. WMA under stress did not correlate with coronary lesion severity, *p* > 0.05. Global longitudinal strain at rest was moderately correlated with the presence of significant coronary artery disease; r = 0.36, *p* < 0.05. Additionally, a moderate correlation exists between global strain under stress and significant coronary artery disease (CAD); r = 0.41, *p* < 0.0001. 

**Figure 3:**
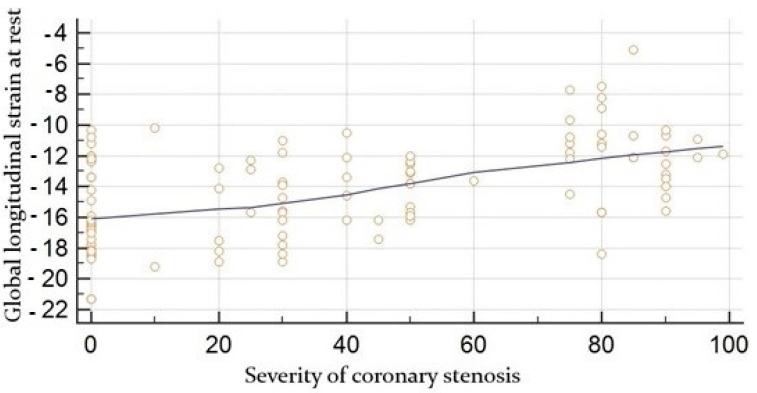
Correlation between global longitudinal strain at rest obtained by speckle tracking (in %) and the angiographic severity of coronary lesion (in %).

**Figure 4:**
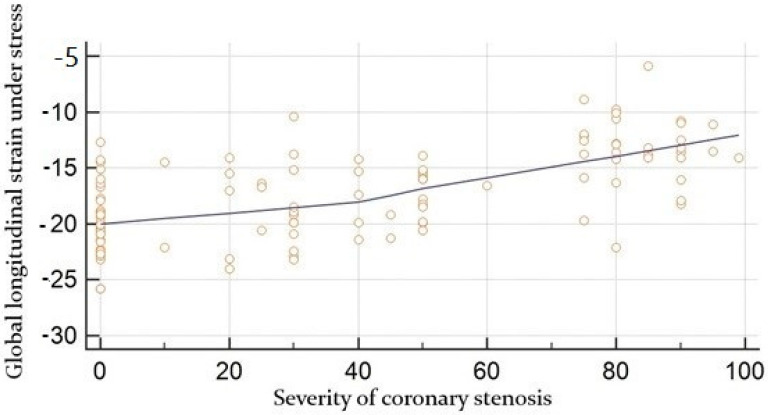
Correlation between global longitudinal strain under stress obtained by speckle tracking (in %) and the angiographic severity of coronary lesion (in %).

The angiographic severity of coronary lesions at rest was moderately correlated with global strain (r = 0.53, *p* < 0.0001) (Figure 3) whereas global strain under stress was moderately correlated with the degree of coronary occlusion, r = 0.62, *p* < 0.0001 (Figure 4).

**Figure 5:**
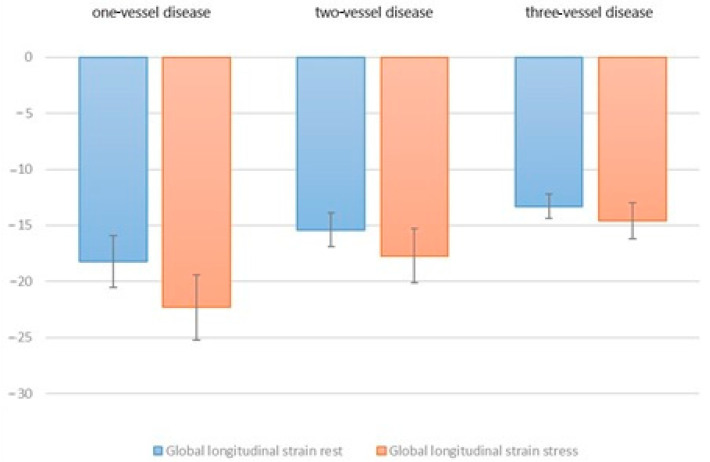
Mean values of global longitudinal strain at rest and under stress in relation to the number of involved coronary vessels.

Under resting conditions, a mean global strain of −18.2% ± 2.3 was detected, and a mean global strain of −22.3% ± 2.9 was detected under stress in patients with one-vessel CAD. In patients with 2-vessel CAD, the mean global strain at rest was −15.4% ± 1.5 and the mean global strain under stress was −17.7% ± 2.4. For patients with three vessels of the coronary artery, the mean global strain was −13.3% ± 1.1 at rest and −14.6% ± 1.6 under stress (Figure 5).

**Figure 6:**
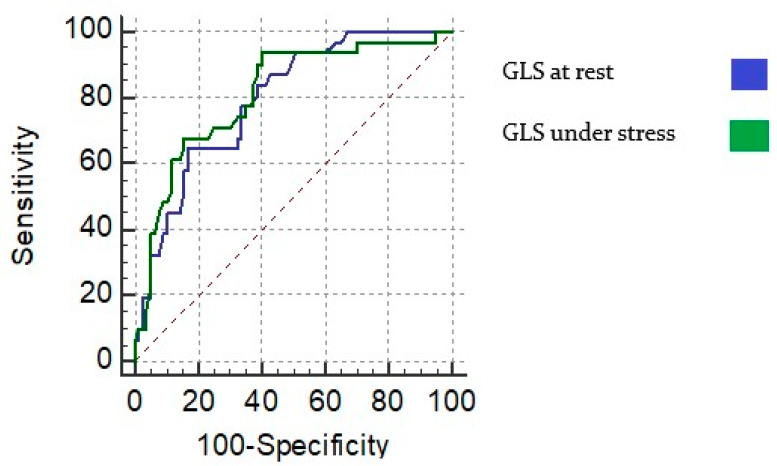
Receiver operating curve for sensitivity and specificity of GLS (global longitudinal strain) at rest and under dobutamine stress in detecting significant coronary artery disease.

A cutoff value of −19.1 (AUC: 0.754) led to a sensitivity of 74.1%, a specificity of 76.7% for detecting significant CAD, with a positive predictive value of 56.1% and a negative predictive value of 88.06% (Figure 6, Table 2).

**Figure 7:**
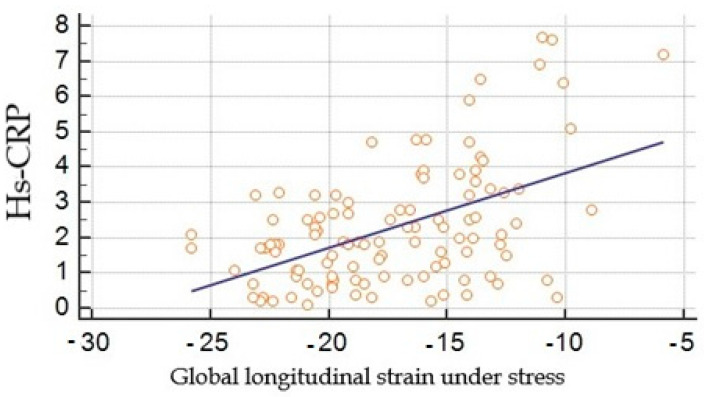
Correlation between global longitudinal strain under stress obtained by speckle tracking (in %) and high sensitivity C-reactive protein (in mg/L).

Hs-CRP correlated with global longitudinal strain parameters at rest and under dobutamine stress, r = 0.4 and 0.5 (Figure 7).

### 3.2. Reproducibility of Obtained Parameters

According to ICC tests, the interobserver agreement was 0.84 for GLS at rest, 0.83 for GLS under stress, and 0.74 for WMA under stress. For the same values, we calculated intraobserver agreement values of 0.85, 0.87, and 0.79, respectively.

## 4. Discussion

A population of patients with suspected CAD presenting in the chest pain unit was investigated for changes in myocardial longitudinal strains at rest and under stress. There is strong evidence that coronary stenosis can affect strain at rest and that longitudinal strain measurements can detect the presence of coronary artery disease [12,13,14]. However, the accuracy of the GLS under stress in diagnosing CAD remains uncertain. GLS is reproducible, regardless of long-term experience in echocardiography [15].

In visual assessment of WMA under stress, a weak correlation with the presence of CAD was observed without significant correlation with the severity of coronary lesions. Our statistical analysis did not include the assessment of resting WMA because of established intermediate sensitivity and specificity, as well as the lack of novelty of such an evaluation against stress-induced WMA.

In both the rest and dobutamine-induced stress conditions, global longitudinal strain was correlated with the occurrence of CAD and with coronary atherosclerosis severity. Further, a cut-off value for GLS under rest and stress was determined based on the number of diseased coronary arteries. Our prospective study showed that a GLS under stress provided better sensitivity and specificity than GLS at rest and the visual assessment of WMA on dobutamine echocardiography.

Few GLS values at rest were lower than −14%. In such patients, we assume the presence of an underlying small vessel disease or microvascular dysfunction which could not be detected in the coronary angiography, but a concomitant peri- or myocarditis cannot be ruled out.

Researchers found that regional 2D strain was reduced in segments supplied by stenotic coronary arteries. A number of studies suggested that impaired longitudinal strains could help identify which coronary artery is stenotic [16,17]. Nevertheless, its diagnostic value has not yet been fully established [18,19]. Based on our current study, we found that GLS is significantly correlated with both the severity of coronary lesion and number of vessels involved, at rest and under pharmacological stress.

The study by dos Santos et al. looked at the applicability of left ventricular longitudinal strain in the emergency room. The authors enrolled 78 patients with clinically suspected unstable angina pectoris. Coronary cineangiography revealed severe coronary lesions in the vast majority of the 15 patients eligible for 2D-STE. Additionally, the authors noticed a significant reduction in global strains in patients with severe lesions in any epicardial coronary artery, as well as a significant reduction in longitudinal strains in the left ventricular inferior and lateral walls of the right and circumflex coronary arteries [20]. In terms of the number of coronary vessels affected, these findings support our current research showing that reduced longitudinal strain is correlated with myocardial ischemia severity.

Another Swedish study that included 296 consecutive patients with clinically suspected stable angina pectoris and normal left ventricular ejection fraction demonstrated the global longitudinal peak systolic strain measured at rest is an independent predictor of significant CAD, and it significantly improved the diagnostic performance of exercise testing. Furthermore, 2D strain echocardiography was able to distinguish high-risk patients [16].

According to a study published in 2018, Scharrenbroich and coauthors assessed differences in strain obtained by speckle tracking and left ventricular ejection fraction to predict cardiac events in patients after acute myocardial infarction, compared to those with known coronary artery disease. By incorporating endocardial GCS (global circumferential strain) to baseline characteristics and ejection fraction into a regression model, ROC analysis significantly enhanced the prediction of cardiac events in patients with CAD (area under curve = 0.86, cutoff value: 20%, sensitivity: 79%, specificity: 84%) [21].

An initial 3D speckle tracking echocardiography was performed on patients with acute coronary syndromes prior to coronary angiography in a cross-sectional study in Bangladesh. Patients with significant stenosis experienced significant reductions in all strain parameters. GLS was demonstrated to effectively identify patients with significant stenosis via receiver operating characteristic curve analysis (area under ROC curve = 0.840, 95% confidence interval = 0.735–0.945). GLS with a cutoff value of −13.50% showed good sensitivity and specificity for detecting significant stenosis [22].

Clinical decision-making could be doubtful in patients without visible regional WMA during pharmacological stress testing and typical angina pectoris symptoms. Strain measurements can provide additional diagnostic information for patients undergoing conventional stress echocardiography [23].

Beyond its sensitive role in diagnosis of infarcted areas in acute myocardial infarction, global longitudinal strain offers important prognostic features. Reduction in GLS could suggest an increased mortality risk, reinfarction, congestive HF, or stroke more reliably than EF and wall motion score index changes [24].

The inflammatory response to early myocardial necrosis is likely to trigger the elevated hs-CRP levels rather than chronic vascular inflammation. In the REGARDS study, CRP was approved as a prognostic indicator for primary prevention for patients with a high risk of cardiovascular disease, defined as Framingham coronary risk score ≥ 10% or atherosclerotic heart disease (ASCVD) risk ≥ 7.5% [25].

In a study of 700 patients with chronic stable angina, serum levels of hs-CRP were strongly associated with the development of cardiac death, non-fatal acute MI, or hospitalization with unstable angina at 1-year follow up [26]. Despite this, it is recommended to combine hs-CRP with other imaging modalities in order to confirm the prognostic effect.

## 5. Limitations

Variability in heart rate had a negative impact on strain rate offline analysis. Many patients were therefore excluded from the study because the analysis was aborted. Atrial fibrillation patients were also excluded due to the inability of the program to compensate for heart rate variability during the study. Furthermore, the failure to obtain a perfect acoustic window during the echocardiography remains a limiting factor among obese patients or patients with hyper-inflated thoracic walls. It can be challenging to capture images with sufficient quality under pharmacological stress. The study is limited by the fact that it is based on only a single center with small number of participants. A large multicenter study is needed to fully corroborate our results. In most cases, coronary occlusion intensity was determined by angiography; only 23 patients were evaluated further using FFR or IFR (instantaneous wave-free ratio). Study and control group ejection fractions were near-normal. Other results could have been obtained with a lower left ventricle ejection fraction group.

## 6. Conclusions

The presence of coronary artery disease can be predicted using longitudinal strain indices at rest and under stress in patients with stable angina pectoris. The results can determine the severity of coronary artery involvement before invasive diagnostic procedures are performed. Stress echocardiography sensitivity and specificity were significantly higher in strain parameters under stress than at rest and in comparison, to traditional visual assessment of wall motion abnormalities in stress echocardiography. Patients with stable angina pectoris should undergo a global longitudinal strain measurement prior to invasive coronary investigation. As an inflammatory marker, hs-CRP can be useful in the acute setting to rule out significant coronary artery disease (CAD), and it should be combined with other imaging modalities to enhance its sensitivity.

## Figures and Tables

**Table 1:** Baseline characteristics based on presence or absence of coronary artery disease (CAD). No: number. %: percentage in each group (no CAD or CAD group). DM: diabetes mellitus. EF: ejection fraction. GLS: global longitudinal strain. Hs-CRP: high-sensitivity C-reactive protein. WMA: wall motion abnormality.

	No CAD	CAD	*p*-Value
Age (years)	65 ± 12	63 ± 9	0.52
Men no.	13	41	0.05
Hypertension no. (%)	27 (77.1)	60 (82.2)	0.35
DM no. (%)	21 (60.0)	31 (42.5)	0.06
Hyperlipidemia no. (%)	12 (34.3)	28 (38.4)	0.42
BMI kg/m^2^	24.7 ± 4.5	25.9 ± 4.2	0.17
Medications			
Aspirin	2 (5.7)	11 (15)	0.17
ACE-Blocker	22 (62)	51 (69)	0.41
Statins	2 (5.7)	3 (4.1)	0.76
Betablocker	6 (17)	38 (52)	0.06
Echocardiographic parameters			
EF (%)	58.0 ± 6.4	48.9 ± 5.9	<0.001
E/A ratio	0.89 ± 0.5	0.89 ± 0.3	0.82
E/E’ ratio	8.1 ± 2.6	8.4 ± 3.2	0.27
E/SRe ratio	0.61 ± 0.45	0.56 ± 0.17	0.29
WMA in stress echocardiography (no.)	31	77	0.006
GLS at rest (%)	−18.4 ± 3.1	−16.9 ± 2.8	<0.001
GLS under stress (%)	−21.9 ± 3.7	−20.8 ± 3.3	0.01
Laboratory findings			
Troponin (ng/mL)	0.068 ± 0.02	0.081 ± 0.07	0.31
Hs-CRP mg/L	1.9 ± 1.5	3.2 ± 1.8	<0.001

**Table 2:** Sensitivity, specificity, positive predictive value, and negative predictive value of visual wall motion abnormalities (WMA) under stress and strain parameters at rest and under stress in detecting CAD. Values in %, 95% CI.

	Visual WMA	Global Longitudinal Strain at Rest	Global Longitudinal Strain under Stress
Sensitivity	61.2 (42.2–78.2)	70.9 (51.9−85.7)	74.2 (55.3–88.1)
Specificity	67.5 (55.9–77.8)	68.8 (57.3–78.9)	76.7 (65.5–85.5)
Positive predictive value	44.2 (33.9–54.9)	47.8 (38.0–57.7)	56.1 (44.7–66.8)
Negative predictive value	81.2 (73.1–87.3)	85.5 (76.9–91.2)	88.1 (80.0–93.1)

## Data Availability

Raw data is available upon request. A public archive was not generated for this study.
